# Effect of a 2-h daily exit on milk quality, activities and outside behavior of lactating cows

**DOI:** 10.3389/fvets.2026.1850312

**Published:** 2026-06-09

**Authors:** S. Arango, L. Bailoni, N. Guzzo, S. Currò, E. Bianco, E. Simonetti, S. Rainis, C. Sartori

**Affiliations:** 1 Department of Comparative Biomedicine and Food Science (BCA), University of Padova, Legnaro, Italy; 2 Regional Agency for the Rural Development (ERSA), Pozzuolo del Friuli, Italy; 3 Department of Agronomy, Food, Natural Resources, Animals and Environment (DAFNAE) University of Padova, Legnaro, Italy

**Keywords:** animal behavior, dairy management, housing systems, milk composition, outdoor access

## Abstract

This study investigated the effect of a 2-h daily exit on milk traits and behavior of dairy cows. A 4-week trial was conducted using 10 lactating cows divided into two homogeneous groups for DIM (129.2 ± 18.8), parity (3.0 ± 1.4) and absence of mastitis and lameness. The experimental design was a crossover (2 groups × 2 periods), so each group was alternatively under treatment IN or OUT in each period. The IN group remained inside a free-stall, while the OUT group had a 2-h daily exit (15:00–17:00) to a short-mowed grass paddock. Milk yield, milk quality, and activities were monitored via individual collars linked to the automatic milking machine. Activities included the number of milkings, feeding (min/d), compound feed intake (kg/d), and rumination (min/d, min/h). Outdoor behaviors were monitored using six digital video cameras covering the paddock area (1,500 m^2^) and connected to a video recorder. Recordings were analyzed by a trained observer. Climate data (relative humidity, temperature, wind speed, solar radiation, rainfall) and the adjusted Temperature-Humidity Index were used to better understand outside behaviors. Statistical analysis followed a mixed model. Results showed a higher daily number of milkings for the IN group (3.02 vs. 2.88; *p* < 0.05), but no differences in milk production (on average 32.6 kg/d) or milk quality traits such as: fat, lactose, and SCS. Milk protein was higher for the IN group (4.81 vs. 4.80%). Eating behaviors were not affected by the treatment with a daily average of 236 min of feeding and 4.53 kg of compound feed intake. Daily rumination was higher for the IN group (485 vs. 472 min), due to the lower rumination of the OUT group during the exit (at 16:00) (19 vs. 7 min) that may have been affected by the grass in the paddock. The main outdoor behaviors were standing (60.45%), grazing (38.10%) and ruminating (11.81%). Rainfall affected (*p* < 0.05) walking, standing, positive interaction, playing and sexual behavior. In conclusion, offering lactating dairy cows a 2-h daily exit in the afternoon is an effective management system, allowing cows to exhibit natural behavior while maintaining milk production and composition comparable to a full indoor system.

## 1 Introduction

Dairy cows have been always reared in many different management systems. Free and tie-stall are indoor systems often used to control feed intake and maximize productivity, from which tie-stall have been always judged for taking away cows the possibility of movement and neglecting animal welfare. On the contrary, extensive systems, where cows stay freely in pasture fields, are often appreciated from the consumer side. Nowadays, mostly due to welfare concerns, mixed systems have arisen as a new trend in dairy cows’ management. A mixed system combines indoor confinement plus an outdoor area that could be used for pasture or just as an exercise area. Providing cows with an outdoor exit seeks to improve animal welfare, as it allows cattle to express normal behavior (1). The use of a pasture area may not be feasible for farmers as grassland areas are not always available. Meanwhile, a paddock used as an exercise area with negligible grass coverage could be easier to provide.

As the dairy industry keeps moving forward on maximizing milk production, automatic milking systems were developed (2). An automatic milking system (AMS) gives cows the free choice to be milked when it better suits them. In order to motivate cows to enter into the milking unit, most of these systems offer concentrates (3). The viability of raising cows in a free stall with an AMS and a paddock as an exercise area has recently been investigated (4). Cows in the paddock no longer have the opportunity to be milked and must wait until they return to the barn. Farmers adopting this type of mixed management may face several challenges, including choosing the appropriate time of day for outdoor access, determining the duration of time cows spend outside, managing feeding schedules, and maintaining milk production and quality. Time spent outdoors should not interfere with cows’ preferred feeding or milking time intervals. If these factors are not well-managed, a daily exit can lead to less feed intake, low milking frequency and reduced milk yield.

Under a mixed management system, dairy cows may change their daily activities and exhibit different behavior patterns inside and outside the stall. The use of an AMS as a technology for precision livestock farming that also monitors individual activities like rumination and eating times could be useful for this purpose. Despite the wealth of research on behavior of dairy cows reared in a free stall, a notable gap remains in understanding how they behave and allocate their activities during a short stay in a paddock. Moreover, the use of a paddock exposes animals to a range of weather conditions including rain, wind and solar radiation, which may also affect behavior and welfare (5). So, this study offers valuable insights into the behavioral time budget of cows during paddock access, in relation to climatic conditions, supporting the development of management strategies that meet animal needs and promote welfare.

The present study evaluated the effect of a 2-h daily exit on milk quality and activities of Italian Simmental lactating cows. An additional objective was to describe outside behaviors and linked them with meteorological parameters.

## 2 Materials and methods

The experimental protocol was evaluated and approved by the ethical committee of the University of Padova (approval number 36/2023) and carried out according to the directive 2010/63/UE of the European Parliament on the protection of animals used for scientific purposes and the Italian law on animal care (Legislative Decree No. 26 of 14 March 2014).

### 2.1 Animals

Ten multiparous lactating Italian Simmental dual-purpose cows were selected from the entire herd of a commercial farm and considered for the trial based on days in milk (129.20 ± 18.83), parity (3.0 ± 1.4), and the absence of mastitis and lameness. They were divided into two balanced groups of five cows. The animals did not have prior experience with outdoor exit, and they ensured the minimum statistical requirement to estimate data variability (6).

### 2.2 Breeding facility and management

**Figure 1:**
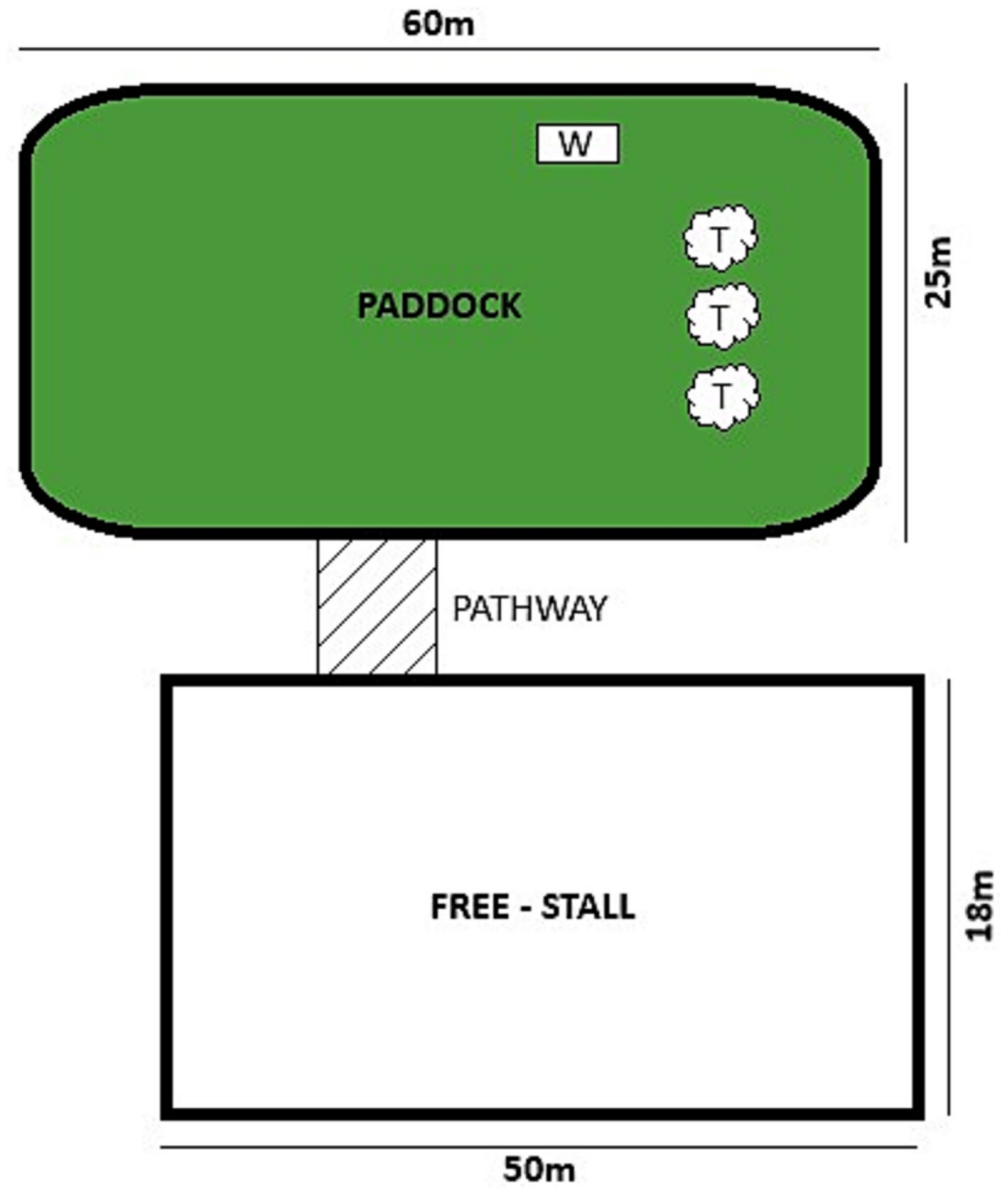
Schematic diagram of the stall and paddock. W, waterer; T, tree.

**Figure 2:**
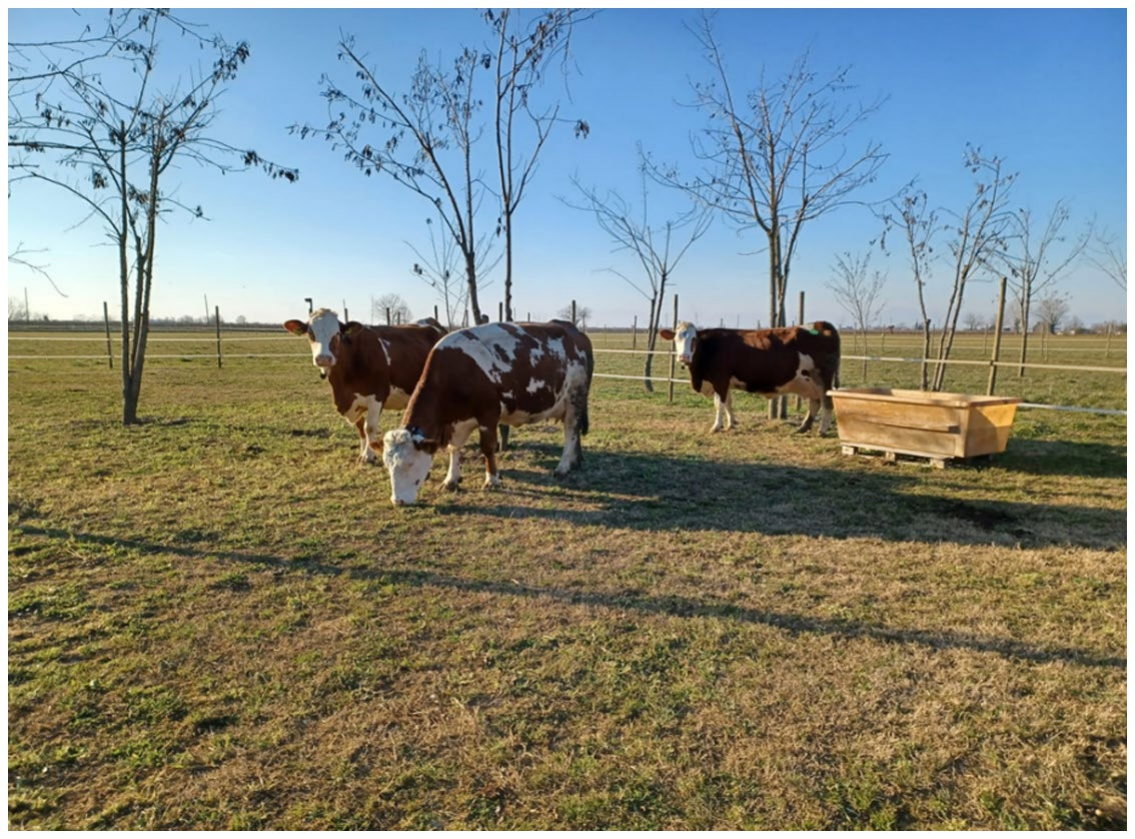
Paddock used for the 2-h daily exit with some cows.

The study was conducted at the “Fattoria di Pavia” farm located in Pavia di Udine (Udine, Italy), and was carried out during the winter season from January 30th to February 26th, 2023, and divided in two consecutive periods of 2 weeks. The breeding facility consisted in a free-stall equipped with an AMS (Lely Astronaut A4, Lely, Maassluis, Netherlands) and an outdoor paddock (Figure 1). The indoor area (18 × 50 m) had concrete flooring and a corridor feeder with available water dispensers. No fans or sprinklers were used. The walking distance to the exercise paddock was 5 m. The outdoor area (Figure 2), encompassing 1,500 m^2^ (60 × 25 m) and surrounded by an electric fence, adhered to the space allowance established by the Royal Society for the Prevention of Cruelty to Animals (7). The paddock had three threes, no shelter and was used just as an exercise area for the animals. Before the experimental period, the paddock grass was trimmed to reduce grazing. Access to the paddock had a duration of 2 h and was allowed 6 days a week.

### 2.3 Diet

In accordance with the guidelines outlined by the National Research Council (8), all animals were fed a total mixed ration (TMR) based on bended alfalfa and alfalfa hay (Table 1) that fulfilled energy requirements for lactating dairy cows. The TMR was distributed once a day during the early morning to the whole herd, so individual intake of TMR was not calculated. TMR samples were collected at the beginning and the end of the experiment, and then analyzed using near-infrared spectroscopy (NIRs) at the LabCNX of the Department of Animal Medicine, Production and Health - MAPS (University of Padova, Legnaro, Italy). Additional compound feed (17.0% CP, 3.8% fat, 7.2% crude fiber, 0.34% sodium, 5.7% ash, expressed as fed) was available during the voluntary access to the AMS and was individually recorded. Fresh water was available ad libitum inside the stall and a water tank with fresh water was available in a corner of the paddock.

**Table 1:** Ingredients and chemical composition of the total mixed ration.

Ingredients	As fed
Bended alfalfa	11.0
Alfalfa hay	6.5
Mixed hay	5.0
Barley meal	3.0
Compound feed^1^	3.0
Water	3.0
Chemical composition	% DM
DM, % as fed	74.08
Crude protein	15.97
Ash	9.51
Lipids	1.40
Neutral detergent fiber	38.76
Acid detergent fiber	25.97
Acid insoluble ash	0.71
Starch	18.87
NE_L_^2^, MJ/kg of DM	5.70

^1^Chemical composition of compound feed: Crude Protein 17.00%. Lipids 3.80%. Crude Fiber 7.20%. Sodium 0.34%. Ash 5.70% as fed. ^2^Net Enery of Lactation according to NRC (8).

### 2.4 Experimental design

The experiment had a duration of 4 weeks divided into two consecutive periods of 2 weeks. Before the beginning of the experiment there was a week of adjustment period for cows to get used to their groups and the paddock. No data was recorded during this week. The study used a changeover experimental design in which the two groups of cows were alternatively exposed to one of the two treatments for 2 weeks and then switched to the other until the completion of the 4 weeks of evaluation. Both treatments were reared in the same free-stall together with the other cows of the herd. Under the IN treatment, the group of cows stayed the whole-day inside the free-stall, whereas, under the OUT treatment cows had a 2-h daily exit (from Monday to Saturday from 15:00 to 17:00). The animals were guided and walked voluntarily until they reached the paddock.

### 2.5 Milk yield and composition

Daily milk production (kg/d) and milk composition (protein, fat, lactose and somatic cell count (SCC)) were individually assessed daily by the AMS.

### 2.6 Activities

Number of milkings (n°/d), feeding time (min/d), compound feed intake (kg/d), and rumination activity (min/d and min/h) were monitored by their individual collars and data was provided by the Qwes System linked to the AMS. Feeding time was predicted based on every time the cows moved their head downwards and was expressed in minutes within a day. The compound feed intake during the stay in the AMS was measured daily and individually along the whole experiment. To better understand the effect of the treatment (IN and OUT) on the rumination activity throughout the day, this parameter was also expressed in minutes per hour.

### 2.7 Outdoor behavioral observations

The outside behavior was monitored using six digital video cameras placed along the two sides of the paddock at a distance of 30 m between each other. A digital video recorder (H.262 Standalone Digital Video Recorder (DVR); Atlantis, Atlantis-land, MI, Italy) was attached to these cameras for continuous recording of behaviors. Individual cows were distinguished by a colored patch attached to their rib side. The recordings were analyzed by a trained observer. Three entire days were lost because of the malfunction of the cameras. As the trial lasted for 4 weeks and cows exited 6 days a week for 2 h, a total of 42 h were evaluated. Behaviors were taken every minute of registration and reported in an Excel spreadsheet. Behaviors (Table 2) were expressed in minutes within each timeslot (2 h).

**Table 2:** Ethogram with behaviors considered during the 2-h exit.

Behavior	Description
Activity behaviors	
Walking	Displacing slowly from one location to another.
Running	Rapid movement with constant changes of direction.
Standing	Standing on four feet, inactive in a relaxed posture; head not moving.
Lying	Resting or sleeping with the legs curled under the body.
Environmental interaction	Sniffing various parts of the stall, floor, objects or sorroundings.
Positive interaction	Staying beside another cow with affiliative postures such as sniffing, smelling and touching gently
Negative interaction	Aggressive actions towards others such as pushing and biting.
Playing	Making physical contact with their body parts, eventually pushing each other without force.
Sexual behavior	Jumping by lifting both forelegs onto the rump of another cow.
Self-grooming	A cow licking any part of itself.
Allogrooming	Grooming and licking another cow using gentle gestures.
Stereotypy	Repetitive or unnatural movement without any apparent function (ex. tongue playing/rolling, bar biting).
Eating behaviors	
Grazing	Moving the head towards the ground trying to grab or bite pasture.
Drinking	Drinking from the water tank.
Ruminating	Chewing motions of teeth while standing, moving or lying.
Excretion behaviors	
Defecating	Elimination of feces standing or moving.
Urinating	Elimination of urine standing or moving.

Climate data used for this study was obtained from the National Aeronautics and Space Administration/Prediction of Worldwide Energy Resources database (NASA/POWER, https://power.larc.nasa.gov) (9). The meteorological parameters considered were: relative humidity, temperature, wind speed, solar radiation, and rainfall. All of them were used to calculate the adjusted Temperature-Humidity Index (THIadj) based on the equation by Mader et al. (10), developed for situations of outdoor monitoring. The THIadj was calculated in every exit and confronted with the seen behaviors during these couple of hours. During the trial, temperatures varied from −0.4 to 10.6 °C, with an average of 6.2 °C and relative humidity that varied from 58.5 to 94.6%, with an average of 77.8%. The minimum daily wind speed was 0.4 km/h and the maximum wind speed recorded was 4.5 mph. The mean of solar radiation was 0.5 and rainfall varied from 0 to 8.7 W/m^2^. The THIadj varied from 32.2 to 55.3 with an average of 46.7.

### 2.8 Statistical analysis

Linear mixed models (MIXED procedure, SAS Institute Inc., Cary NC, 2014) were run to interpret the data collected assuming a normal distribution of the variables.

Milk traits and cow activities were analyzed using the following mixed model:


$$Y_{\text{ijklmno}}=µ+T_{i}+P_{j}+{\left(T^{*}P\right)}_{\mathrm{ij}}+{\mathrm{DIM}}_{k}+L_{l}+{\mathrm{day}}_{m}+{\mathrm{cow}}_{n}+e_{\text{ijklmno}}$$

where Y is the trait, μ is the general mean, T is the fixed effect of the treatment (IN and OUT), P is the fixed effect of the period (2 levels), T * P is the interaction between the treatment and the period, DIM is the fixed effect of the days in milk (5 levels: defined every 20 DIM starting from the calving day), L is the fixed effect of the number of lactation (3 levels), day is the random effect of the date at recording (starting from 1, which corresponds to the 30th of January 2023), cow is the random effect of the individual, and e is the residual error.

The statistical model applied for the hourly rumination activity was:


$$\begin{array}{c} Y_{\text{ijklmnop}}=µ+H_{i}+T_{j}+P_{k}+{\left(H^{*}T\right)}_{\mathrm{ij}}+{\left(T^{*}P\right)}_{\mathrm{jk}}+{\mathrm{DIM}}_{l}+L_{m}\\ +{\mathrm{day}}_{n}+{\mathrm{cow}}_{o}+e_{\text{ijklmnop}} \end{array}$$

including the same effects of the previous model plus the fixed effect H of the hour of the day (24 levels).

The analysis of outdoor behaviors focused on detecting possible climate parameters influencing their expression during the exit. Therefore, a biologically informed variable selection approach with correlation screening was applied to choose which effects to include in the model. The base model included only the fixed effect of the period (2 levels), and then was modified to also consider the variability due to the study subjects and the period of observation. Climate variables (temperature, humidity, wind, solar radiation and rainfall) were included as effects in the model by only keeping the ones with biological relevance while excluding highly correlated predictors based on Pearson correlation coefficients. All climate variables were included as covariates except for rainfall, handled as a fixed effect with three rain classes (no rain, moderate, high) due to its non-normal distribution. About 10 different models were tested for each behavior following this procedure, plus a model able to account for all of them in terms of adjusted Temperature-Humidity Index (THIadj) and rainfall classes. The best model for each behavior was chosen based on the Akaike Information Criterion (AIC; (11)) and at the *F*-values of the ANOVA fixed effect testing of the linear mixed model. Therefore, the statistical model applied for outdoor behaviors was:


$$\begin{array}{c} Y_{\text{ijklmnopq}}=µ+P_{i}+[{\text{THIadj}}_{j}+T_{k}+H_{l}+W_{m}+S_{n}+R_{o}]+\mathrm{day}{\left(P\right)}_{\mathrm{ip}}\\ +\mathrm{cow}{\left(P\right)}_{\mathrm{iq}}+e_{\text{ijklmnopq}} \end{array}$$

where Y is the target behavior, *μ* is the general mean, P is the fixed effect of the period or group of cows (2 levels), THIadj is the fixed effect covariate of the adjusted Temperature-Humidity Index, T is the temperature covariate, H is the humidity covariate, W is the wind speed covariate, S is the solar radiation covariate, R is the fixed effect of the rainfall (3 levels: no rain, low rain, high rain), day is the random effect of the date within P, cow is the random effect of the cow within P as well, and e_ijk_ is the residual error. Climate effects in brackets shows that only some of them were included in the final model for each behavior depending on the AIC and *F* statistics. The effects of day and cow were included within P because each one of their levels were fully included within one of the levels of P. All main effects were kept in the model at the beginning regardless of the significance level.

As a post-hoc analysis, the Least Square Means for the levels of the fixed effects were computed and compared using a Student’s *t*-test. For all the analyses, a *p*-value < 0.05 was considered to assess the statistical significance, whereas a *p*-value < 0.1 was reported as a suggestive significance.

## 3 Results

Results of the mixed model analysis on milk traits and animal activities are reported in Table 3.

**Table 3:** Least square means (LSM) of the fixed effects and ANOVA (*F*-value) of milk traits and activities.

Parameter	LSM			ANOVA (*F*-value)				
	IN	OUT	SE	*T*	*P*	*T* × *P*	DIM	*L*
Milk traits								
Milk yield, kg	32.69	32.50	1.34	0.89	2.72°	0.26	6.15^**^	0.32
Protein, %	4.81^a^	4.80 ^b^	0.02	4.03^*^	5.91^*^	2.33	2.13°	2.18
Fat, %	3.83	3.77	0.34	3.05°	0.01	0.16	6.31^**^	2.23
Lactose, %	4.81	4.80	0.02	3.49°	7.18^**^	1.88	2.39°	2.60°
Protein, kg	1.13	1.12	0.04	2.57	13.05^**^	0.18	6.20^**^	0.27
Fat, kg	1.25	1.23	0.09	2.51	0.46	0.02	7.92^**^	4.10^*^
Lactose, kg	1.57	1.56	0.06	1.24	1.73	0.14	7.03^**^	0.62
SCS^1^	3.32	3.29	0.51	0.19	0–0.03	0.23	2.88^*^	1.40
Activities								
N° milkings, n/d	3.02 ^a^	2.88 ^b^	0.20	3.77^*^	0.00	1.35	1.28	0.75
Feeding, min/d	234.4	237.6	20.14	0.55	0.82	2.18	6.64^**^	0.35
Feed intake, kg/d	4.54	4.51	0.17	0.12	1.39	1.96	2.38^*^	1.10
Rumination, min/d	485.2^a^	472.0^b^	17.59	5.13^*^	0.72	0.07	0.81	0.03

IN, no daily exit; OUT, 2-h daily exit; SE, Standard Error; *T*, Treatment; *P*, Period; *T* × *P*, Treatment × Period; DIM, Days in milk, *L*, Lactation number. ^1^Calculated as 3 + log_2_(SCC/100,000). ^a,b^Means with different superscript letters are statistically different. ^*^p < 0.05. ^**^p < 0.01. °*p* < 0.10.

### 3.1 Milk parameters

The average daily milk production was 32.60 kg/d and no significant overall differences between the two treatments were observed. Milk composition was similar for both treatments, except for the percentage of protein, which was higher in group IN (4.81 vs. 4.80%; *p* < 0.05). In average, milk had 3.80% fat, 4.81% lactose, 1.13 kg protein, 1.24 kg fat, 1.57 kg lactose, and 3.30 SCS. All the milk traits were affected by the days in milk (*p* < 0.05).

### 3.2 Animal activities

**Figure 3:**
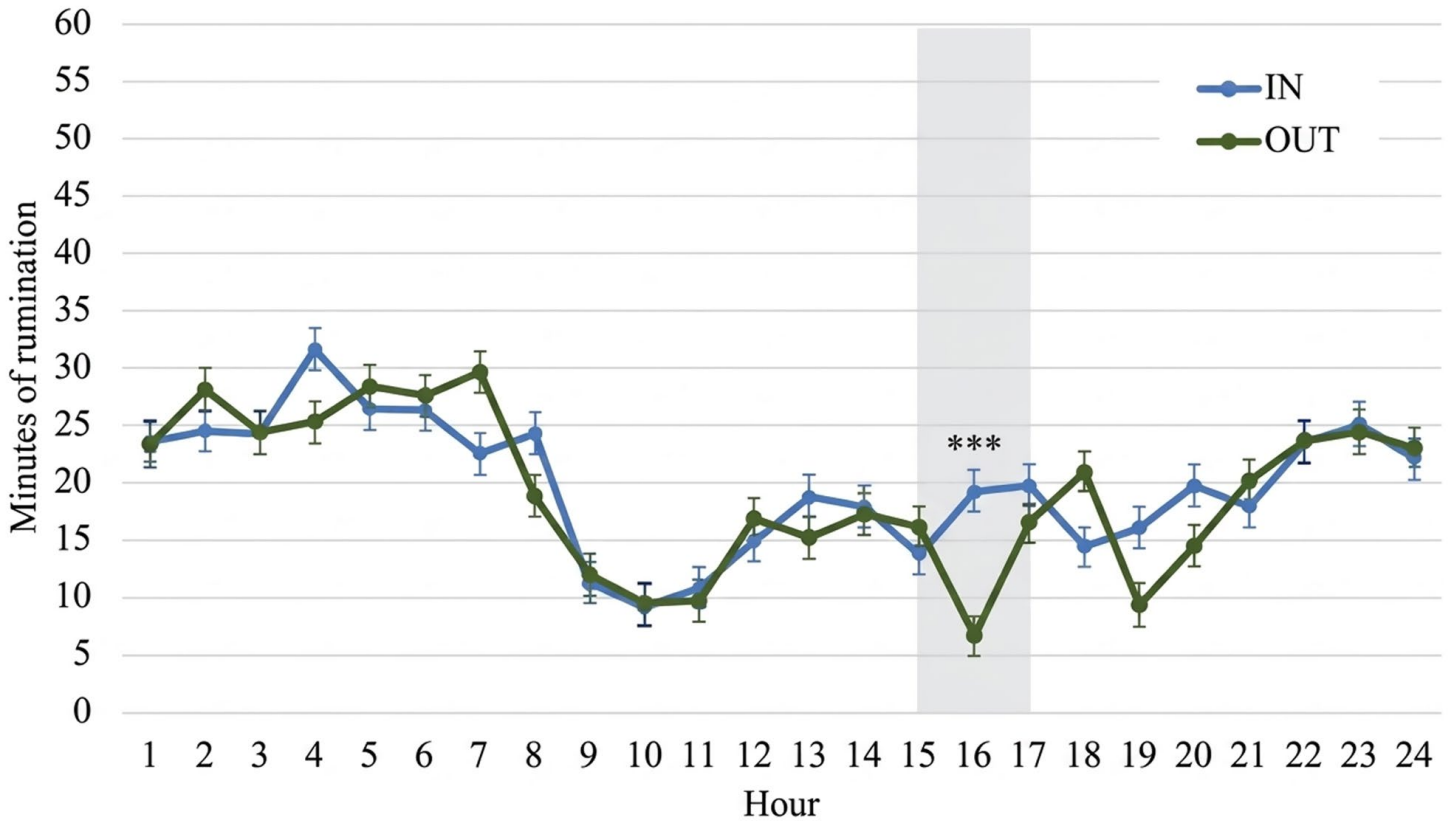
Daily rumination activity (LSM ± SE) of the indoor (IN) and the outdoor group (OUT) with a 2-h daily exit (grey bar) during the experimental period. ^***^Means are statistically different *p* < 0.001.

The daily number of milkings was higher for the IN group (3.02 vs. 2.88; *p* < 0.05). Feeding behavior parameters, such as feeding time and feed intake were not affected by the treatment. In average, daily feeding time was 236.04 min and compound feed intake was 4.53 kg/d. The time of daily rumination was higher for the IN group (485.2 vs. 472.0 min; *p* < 0.05). The daily trend of the rumination activity of the two treatments is represented in Figure 3. During hour 16, corresponding to the time which OUT cows were in the paddock and IN cows were inside the stall, rumination was higher for the IN group (19.26 vs. 6.69 min; *p* < 0.01).

### 3.3 Outdoor behavior pattern

**Figure 4:**
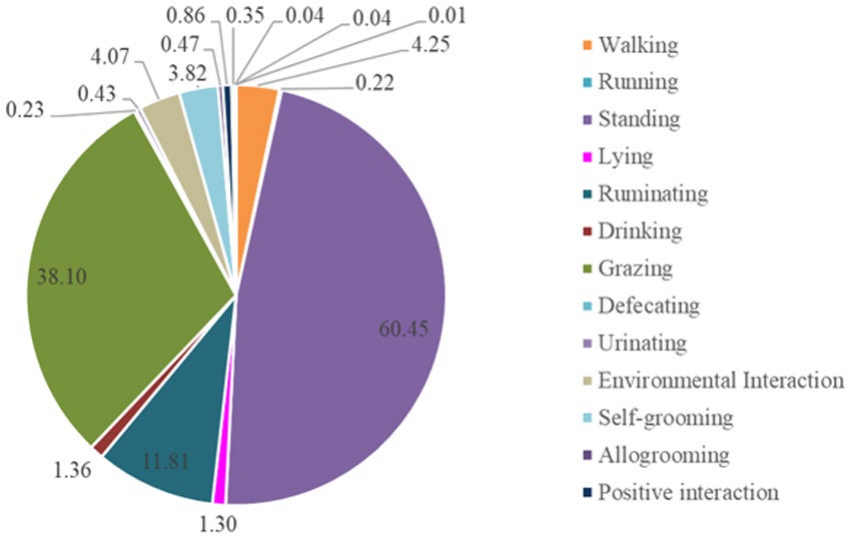
Distribution (%) of the outdoor behaviors during the 2-h exit.

Behaviors observed during the 2 h spent in the paddock are represented in Figure 4. The main outdoor behaviors were standing (60.45%), grazing (38.10%) and ruminating (11.81%).

Results of the mixed model of the outside behaviors are displayed in Table 4. The THIadj had almost no effect on the outdoor behaviors, just walking resulted to be significantly affected (*p* < 0.05) by this index (data not shown). After choosing the best model for each behavior, it was possible to identify the climate parameters that most affected each of them individually. Only two behaviors (environmental interaction and positive interaction) differed (*p* < 0.05) between periods (group of cows). Meanwhile, rainfall affected (*p* < 0.05) walking, standing, positive interaction, playing and sexual behavior. The presence of rain increased the time spent walking, doing positive interaction and sexual behavior, but decreased the time standing and playing. Little influence of other climate effects was found. As the temperature increased, walking (*p* < 0.05) increased but a slight decrease in running (*p* < 0.01) and positive interactions (*p* < 0.05) were seen. As wind got stronger, self-grooming (*p* < 0.05) and urination (*p* < 0.01) increased, and negative interactions decreased (*p* < 0.01). When solar radiation was higher, playing behavior (*p* < 0.05) decreased.

**Table 4:** Type III test of fixed effects for the selected outside behavior models.

Trait	Effect	*F*	*P*	*b*	SE*_b_*	LSM^1^	LSM^2^	LSM^3^	SE_LSM_
Activity behaviors									
Walking	Period	4.31	°	–	–	7.19	3.15	–	2.26
	Rain	4.25	^*^	–	–	3.10^b^	5.32^a^	7.09^a^	4.82
	Temperature	4.84	^*^	0.62	0.28	–	–	–	–
	Wind	3.50	°	−1.73	0.92	–	–	–	–
Running	Period	3.67	°	–	–	−0.32	0.71	–	0.53
	Rain	1.44	ns	–	–	−0.15	0.37	0.38	0.73
	Temperature	8.62	^**^	−0.26	0.09	–	–	–	–
	Humidity	3.63	°	−0.03	0.02	–	–	–	–
Lying	Period	0.22	ns	–	–	−0.69	1.74	–	1.29
	Rain	0.44	ns	–	–	1.29	1.42	−1.15	1.46
	Wind	0.98	ns	−0.03	1.22	–	–	–	–
	Solar radiation	0.92	ns	0.85	7.90	–	–	–	–
Standing	Period	0.29	ns	–	–	64.7	58.0	–	8.92
	Rain	3.47	^*^	–	–	67.9^a^	54.5^b^	61.7^ab^	43.6
Environmental Interaction	Period	13.93	^**^	–	–	7.93^A^	1.52^B^	–	1.50
	Rain	1.25	ns	–	–	2.72	5.95	5.51	4.67
	Wind	0.15	ns	−0.58	1.47	–	–	–	–
	Solar radiation	1.07	ns	9.86	9.54	–	–	–	–
Positive interaction	Period	11.42	^**^	–	–	0.14^B^	2.20^A^	–	1.33
	Rain	6.47	^**^	–	–	0.02^C^	0.97^B^	2.53^A^	1.54
	Temperature	4.13	^*^	−0.22	0.11	–	–	–	–
	Solar radiation	0.41	ns	1.48	2.31	–	–	–	–
Negative interaction	Period	0.49	ns	–	–	0.27	0.48	–	0.36
	Rain	1.24	ns	–	–	0.05	0.57	0.50	1.21
	Wind	7.92	^**^	−0.67	0.24	–	–	–	–
	Solar radiation	0.54	ns	1.13	1.53	–	–	–	–
Playing	Period	0.19	ns	–	–	0.04	0.00	–	0.04
	Rain	3.69	^*^	–	–	0.14^a^	−0.04^b^	−0.04^b^	−0.20
	Wind	0.5	ns	0.03	0.05	–	–	–	–
	Solar radiation	5.79	^*^	−0.75	0.31	–	–	–	–
Sexual behavior	Period	0.22	ns	–	–	0.13	0.10	–	0.08
	Rain	5.35	^**^	–	–	0.00^B^	0.02^B^	0.33^A^	0.13
	Wind	0.07	ns	0.02	0.06	–	–	–	–
	Solar radiation	0.97	ns	0.38	0.39	–	–	–	–
Self-grooming	Period	0.05	ns	–	–	3.90	4.27	–	2.76
	Rain	0.42	ns	–	–	3.63	3.71	4.92	3.81
	Wind	4.05	^*^	1.65	0.82	–	–	–	–
	Solar radiation	0	ns	−0.37	5.32	–	–	–	–
Allogrooming	Period	0.27	ns	–	–	0.74	0.51	–	0.44
	Rain	0.94	ns	–	–	0.58	0.29	1.00	0.65
	Wind	0.04	ns	−0.07	0.34	–	–	–	–
	Solar radiation	0.21	ns	1.01	2.21	–	–	–	–
Stereotypy	Period	0.15	ns	–	–	0.01	0.00	–	0.02
	Rain	3.04	°	–	–	0.05	−0.02	−0.02	0.02
	Wind	1.32	ns	0.03	0.02	–	–	–	–
	Solar radiation	3.70	°	−0.28	0.15	–	–	–	–
Eating behaviors									
Grazing	Period	0.21	ns	–	–	32.0	39.2	–	25.27
	Rain	0.42	ns	–	–	38.0	38.7	30.0	27.30
	Temperature	3.00	°	2.98	1.72	–	–	–	–
	Solar radiation	1.01	ns	−36.80	36.54	–	–	–	–
Drinking	Period	0.28	ns	–	–	1.75	8.65	–	0.30
	Rain	2.55	°	–	–	1.41^ab^	1.14^b^	2.40^a^	2.41
Ruminating	Period	0.85	ns	–	–	14.17	10.27	–	2.62
	Rain	1.38	ns	–	–	14.85	9.57	12.24	2.75
	Temperature	0.17	ns	0.29	0.71	–	–	–	–
	Wind	1.22	ns	2.68	2.43	–	–	–	–
	Solar radiation	1.77	ns	−20.98	15.78	–	–	–	–
Excretion behaviors									
Defecating	Period	0.01	ns	–	–	0.24	0.26	–	0.21
	Rain	0.11	ns	–	–	0.23	0.21	0.31	0.85
	Temperature	1.25	ns	0.05	0.04	–	–	–	–
	Solar radiation	3.47	°	−1.62	0.87	–	–	–	–
Urinating	Period	0.13	ns	–	–	0.51	0.43	–	0.30
	Rain	1.38	ns	–	–	0.54	0.32	0.55	1.21
	Temperature	1.28	ns	0.04	0.04	–	–	–	–
	Wind	10.46	^**^	0.40	0.12	–	–	–	–
	Solar radiation	3.03	°	−1.39	0.80	–	–	–	–

*F*, *F*-value; *p*, *p*-value; *b*, coefficient of regression; SEb, standard error of b; LSM, least square means of the period (LSM^1^: for period 1. LSM^2^: for period 2) or the level of rain (LSM^1^: no rain, LSM^2^: low rain, LSM^3^: high rain). SE_LSM_: standard error of the LSM. ^*^p < 0.05. ^**^p < 0.01. °*p* < 0.10. ns, not significant. ^a,b, A, B^Means with different superscript letters are statistically different.

## 4 Discussion

### 4.1 Milk parameters

Many farmers believe that dairy cows will not maintain their milk production when they stayed in an outside area and the use of automatic milking systems adds an extra challenge to this (1). Considering recent scientific studies conducted in Italy using Italian Simmental dairy cows, the average milk yield of this breed has been reported to be 22.0, 25.5 and 28.46 kg/d (12–14). In this experiment, cows reared indoors and cows that had a 2-h daily exit produced 32.69 and 32.50 kg/d, respectively. The time cows spent outside the stall could be seen as a waste of time for farmers because it could decrease the time cows spent eating, meaning less feed intake and consequently a decrease in milk production. This assumption could be refused by this experiment that demonstrated that milk production was not affected when cows stayed 2 h a day in an outside area. The same was found on a 6-month trial, where lactating Swedish Red and White dairy cows maintained their milk yield after staying 1 h per day (13:00–14:00) in a paddock used as an exercise area (15).

Fat, lactose and SCS were consistent with the chemical composition of milk coming from Italian Simmental dairy cows raised in Italy (12–14). Protein, an important component of milk, resulted higher in the IN group but only in terms of percentage and not as quantity.

### 4.2 Activities

The number of daily milkings is a significant predictor of milk yield (16), with a positive correlation between the two of them (17). Despite the similar milk yield between the two treatments, OUT cows had a lower number of milkings than IN cows. It seems likely that the 2 h spent outside without the possibility of accessing the AMS is the main explanation for this difference. With 2 h less inside the stall, the number of milkings was meant to be reduced. As this reduction did not affect the overall milk yield, it seems that cows were able to maintain their daily milk production even with 0.14 less milkings a day. The timeslot chosen (15:00–17:00) for the exit must have contributed to this, because it might not be among the timeslots cows prefer to be milked. In fact, a study showed that the average percentage of cows in the milking robot between the 15:00 and 17:00 h were below the average of 2% of the whole-day (16). Moreover, the duration of the exit also seems to be crucial. As just 2 h were enough to reduce significantly the daily number of milkings, a longer duration could not be able to hold the milk production and will be detrimental. It would be valuable if further studies could be conducted to determine the optimal duration of the exit. A pilot study performed in an experimental farm of Simmental cows, suggested that a 4-h daily outdoor access split into two timeslots (2 h in the morning and two in the afternoon) was favourable for cows, but for managing reasons also a single exit of 2 h offers a valuable solution (18).

Surprisingly, feeding parameters measured in this study were not affected by the treatment. The fact that cows remained outside for 2 h could have wrongly suggested that as they had been taken away the possibility of access into the barn and therefore into the AMS, they will also decrease their daily feeding time and their compound feed intake. But this experiment showed that just a couple of hours without being able to access the AMS is considered as a short period that did not affect cows’ eating behavior. Moreover, this was supported by earlier findings where the daily eating time of dry Holstein cows was similar between a group raised in a shed indoors and a group raised in a shed with access to an exercise paddock (19). Unfortunately, TMR intake was not possible to calculate in order to make any further discussion.

The time cows spent ruminating in both treatments showed a normal daily pattern (20, 27), but the difference between both treatments was clear. Rumination, as a voluntarily controlled behavior, resulted to be different at hour 16:00 during the timeslot where OUT cows were in the paddock and IN cows were inside. Meanwhile, when both groups were inside the stall, rumination activity was not different between them and followed the normal pattern of the breed (20). This result enhances the effect of the location, as our findings revealed a reduced duration of rumination when cows spent 2 h outdoors. Literature suggests that animals will stop ruminating when they are disturbed (21), and cows could have felt disturbed when they were conducted to the paddock. Moreover, the small presence of grass in the paddock could have interfered with rumination. Even though the grass of the paddock was trimmed, grazing was the second most seen outdoor behavior and cows do not ruminate when they graze.

### 4.3 Outdoor behaviors

The type of mixed management system used in this study could be seen as a physical environmental enrichment that enhances animal welfare. While indoors the use of the AMS itself is already considered a strategy for this type on enrichment (22, 23), the use of a paddock as an external area for a short stay could fit in this definition too. The possibility of choosing outdoors was not given to the cows in this experiment, meaning that the outdoor access was set as a fixed routine for them.

Besides new insights on mixed managements systems, this study uniquely provides the behavioral pattern of dairy cows in a paddock linked with climate parameters. Cattle have a thermoneutral zone, which ranges between 2 and 25 °C for lactating dairy cows (5). In this trial, cows in the paddock were in good thermal comfort because the THIadj did not surpass the maximum of 72 and only 3 days were slightly below the thermoneutral zone. The fact that cows were not able to choose to come back to the barn if they wanted to, allowed us the possibility to identify the climatic parameters that had most impact on outside behavior. According to our observations, cows that had a 2-h daily exit seemed to express their natural behavior when outdoors.

Standing was the most seen behavior in the paddock, which is consistent with the literature (4, 15, 18, 24). Instead of walking around the new area, cows preferred standing. Apparently, standing is a behavior that would always be the main activity when cows spend some time in a paddock. In fact, the behavior pattern of dairy cows that spent 1 h per day in a paddock consisting of clay soil covered with little grass and used only as an exercise area, showed that standing was the number one seen behavior covering 31% of their time-budget (15). This might relate to the preference of dairy cows to rest standing when they are in a grass covered soil as it represents its ideal flooring (25). Due to the fact that the only climatic parameter that reduced the time cows spent standing was rainfall, this could suggest to avoid rainy days when this type of management is used.

Although the second most seen behavior was grazing, this could not be considered as effective grazing and just as a graze attempt because grass in the paddock was previously trimmed. This shows that if cows stay in a grass covered area, they will tend to graze, and could be linked with their natural behavior in pasture. The same was seen in other experiments where cows put their muzzles down expressing grazing behavior even in an area with grass cut to ground level (1, 5). Climatic parameters seemed not to affect grazing behavior during the stay in the paddock.

Rumination, one of the most important behavioral traits in ruminants, was the third most seen behavior during the outside stay. It reflects a good welfare state, but it is generally affected by a lot of factors such as feed quality and composition, health status, stocking rate, grouping, etc. (21). The similar time of the two groups of cows doing rumination while they were in the paddock along with the lack of difference of the period and climatic conditions suggests that rumination remained stable during the exit. So, even though there was a clear reduction in the time spent ruminating when cows were outside (Figure 3), this reduction was seen in both groups of cows and in different periods which basically demonstrated that the exit disturbed the cows leading to a collective and marked decrease of this behavior in the paddock. Moreover, the limited grass available in the paddock, which allowed some grazing activity, may have contributed to the reduction in rumination time as cows do not ruminate while grazing.

Stereotypies and negative interactions were basically not observed or observed just in a very few occasions in both periods, showing a positive welfare status of the cows. Normally, stereotypies like tongue rolling are not observed on pasture (4) and even though this experiment did not offer the possibility of effective grazing, the fact that some grass was available seemed to be well appreciated by cattle. The little time cows spent doing negative interaction and the lack of difference between both periods suggests that both groups might have been fully adapted to each other, that the week of adaptation period was enough for cows to build hierarchy, so they did not see each other as opponents during the exit.

Walking was an interesting behavior to see as it was affected by the period (group of cows), the THIadj, rainfall, temperature, and wind. The disaggregation of the THIadj showed that the main climatic parameter affecting walking was temperature, in agreement with the literature (26). Even though this trial was conducted in winter with low temperatures and inside the thermoneutral zone of the cows, a raise in temperature and the presence of rain increased the time cows spent walking in the paddock. This could mean that walking was elicited not only by the change of the environment (from confinement to the external area), but also by rain and temperature as the two main external stimuli. Cows may have increased their time walking to show discomfort and search for shelter during rain or when the temperature raised.

The space allowance in the paddock needs to be considered as an important factor in the whole behavior pattern of dairy cows. For this trial, the paddock allowed a space of 300 m^2^ per cow which is almost 6.7 times more than the space they had inside the stall. The space allowance is inversely correlated with social interaction behavior, as the more space you give to the animal will reduce interactions between them (4). In fact, during the 2 h in the paddock, cows spent little time on social behaviors such as playing or allogrooming. Also, a high outdoor space allowance increases the time standing (4) which indeed was the most seen behavior in the paddock.

The differences found for some of the outside behaviors (walking, running, environmental and positive interactions) between the two periods should be seen as the differences between the two groups of cows because each period corresponded to each group of animals. Weather during the winter period was stable during the trial, it always showed low temperatures and rainfall occurred sporadically. On one hand, gathering the climatic parameters to calculate the THIadj was not able to explain outside behaviors, as this index only affected the time spent walking in the paddock. In addition, using the composite index did not allow the determination of possible effects of each different climate variable. On the other hand, rainfall had more impact as it had an effect on walking, standing, positive interaction, playing and sexual behavior. Actually, it was already seen that cows did not prefer to stay under the rain when outdoors (4). This suggests that providing some natural or artificial shade during hot and wet weather should be considered to reduce discomfort in cows. Despite all this, in general, cows displayed the same behavior pattern in both periods when outdoors, suggesting a consistency of behaviors when different cows were outside.

Concluding, the study found a consistency of milk production and animal activities for dairy cows kept only indoors or experiencing 2 h of daily exit, with the exception for rumination that decreased during the stay in the paddock. The outdoor behavior pattern was consistent between the different groups of cows and seem to be scarcely affected by climate variables during the winter period. Additional studies could help to better understand the effects of an outdoor exit on the daily activity of dairy cows. Results of this study could already offer some practical indications for this purpose, suggesting that an outdoor exit is promising for enhancing cows’ welfare.

## Data Availability

The original contributions presented in the study are included in the article/supplementary material, further inquiries can be directed to the corresponding author.
